# Activity-Dependent Modulation of Odorant Receptor Gene Expression in the Mouse Olfactory Epithelium

**DOI:** 10.1371/journal.pone.0069862

**Published:** 2013-07-29

**Authors:** Shaohua Zhao, Huikai Tian, Limei Ma, Ying Yuan, C. Ron Yu, Minghong Ma

**Affiliations:** 1 Department of Neuroscience, University of Pennsylvania Perelman School of Medicine, Philadelphia, Pennsylvania, United States of America; 2 Department of Geriatric Cardiology, Qilu Hospital of Shandong University, Jinan, Shandong, China; 3 Stowers Institute for Medical Research, Kansas City, Missouri, United States of America; Duke University, United States of America

## Abstract

Activity plays critical roles in development and maintenance of the olfactory system, which undergoes considerable neurogenesis throughout life. In the mouse olfactory epithelium, each olfactory sensory neuron (OSN) stably expresses a single odorant receptor (OR) type out of a repertoire of ∼1200 and the OSNs with the same OR identity are distributed within one of the few broadly-defined zones. However, it remains elusive whether and how activity modulates such OR expression patterns. Here we addressed this question by investigating OR gene expression via in situ hybridization when sensory experience or neuronal excitability is manipulated. We first examined the expression patterns of fifteen OR genes in mice which underwent neonatal, unilateral naris closure. After four-week occlusion, the cell density in the closed (sensory-deprived) side was significantly lower (for four ORs), similar (for three ORs), or significantly higher (for eight ORs) as compared to that in the open (over-stimulated) side, suggesting that sensory inputs have differential effects on OSNs expressing different OR genes. We next examined the expression patterns of seven OR genes in transgenic mice in which mature OSNs had reduced neuronal excitability. Neuronal silencing led to a significant reduction in the cell density for most OR genes tested and thinner olfactory epithelium with an increased density of apoptotic cells. These results suggest that sensory experience plays important roles in shaping OR gene expression patterns and the neuronal activity is critical for survival of OSNs.

## Introduction

The mammalian olfactory system utilizes a large family of odorant receptors (ORs) for detecting numerous odors in the environment. The mouse olfactory epithelium contains several million ciliated olfactory sensory neurons (OSNs), each of which expresses a single OR type out of a repertoire of ∼1200 [1]. Smell perception starts with binding of odor molecules to specific ORs, which triggers a second messenger cascade leading to opening of specific ion channels in the activated OSNs. The subsequent depolarization of the cell membrane generates action potentials, which carry the odor information into the olfactory bulb [2], [3], [4]. The OSNs expressing a particular OR are scattered in one of the few broadly-defined zones [5], [6], [7], [8], while their axons typically converge onto two discrete glomeruli in the olfactory bulb [9].

The olfactory epithelium is continuously regenerating throughout life. OSNs can undergo caspase-mediated apoptosis (programmed cell death) at all stages in their life cycle and be replenished by newly generated OSNs from dividing basal cells to maintain the epithelial homeostasis [10], [11], [12]. This makes the olfactory system especially subject to activity-dependent modulation. Unilateral naris closure has been widely used to manipulate sensory inputs into the two nostrils of a rodent: the closed side is deprived of most airflow and odor stimulation, whereas the open side experiences greater than normal stimulation. This procedure causes structural, molecular, and functional changes in both the closed and open side [13], [14], [15], [16], [17], [18], [19], [20], [21], [22], [23], [24]. In addition, spontaneous and odor-induced neuronal activity plays critical roles in survival and proper targeting of OSNs [25], [26], [27], [28], [29], [30]. However, it remains elusive to what extent sensory experience and neuronal activity modulate the expression patterns of OR genes in the olfactory epithelium.

In the current study, we first investigated the expression patterns of selected OR genes using *in situ* hybridization in mice which underwent neonatal, unilateral naris closure. After four-week occlusion, the expression patterns of fifteen OR genes showed different changes. The cell density in the closed side was significantly lower (for four ORs), similar (for three ORs), or significantly higher (for eight ORs) as compared to that in the open side, suggesting that sensory inputs have differential effects on OSNs expressing different ORs. We then investigated how decreased neuronal excitability would affect OR gene expression patterns in the transgenic mice in which olfactory marker protein (OMP) drives overexpression of the inward rectifying potassium channel (Kir2.1) in mature OSNs to hyperpolarize the membrane [25]. The cell density for six out of seven OR genes tested was significantly lower in OMP-Kir2.1 mice as compared with the wild-type controls. In addition, OMP-Kir2.1 mice had thinner olfactory epithelium with an increased density of apoptotic cells, supporting the notion that neuronal activity is critical for survival of OSNs. The results suggest that sensory stimulation and neuronal activity play distinct roles in shaping the expression patterns of OR genes in the olfactory epithelium.

## Materials and Methods

### Tissue Preparation

Wild-type C57Bl/6 mice were purchased from the Jackson Laboratory. For unilateral naris closure, a brief cauterization (<1 s) with a cauterizer (Fine Science Tools, Foster City, CA, USA) was performed on one nostril at postnatal day 3 (P3), and the mice were examined 28 days later at the age of one month. Only mice with complete closure on the operated side were used for further analysis. OMP-Kir2.1 mice and their littermate controls (one month old) were obtained by crossing two genetically-modified lines: (1) OMP-IRES-tTA mice in which OMP positive cells also express the transcriptional activator tTA directed by an internal ribosome entry site (IRES), and (2) tet_o_-Kir2.1-IRES-taulacZ mice in which the tTA-responsive promoter tet_o_ drives the bicistronic expression of the inward rectifying potassium channel Kir2.1 and the marker taulacZ [25]. These transgenic mice were rederived and backcrossed in C57Bl/6 background. The animal number for each condition is as follows: naris-closed (12), wild-type (12), OMP-Kir2.1 (8), and littermate controls (6).

Mice were deeply anesthetized by intraperitoneal injection of ketamine/xylazine (200 mg/15 mg/kg body weight) before decapitation. The heads were fixed in 4% paraformaldehyde (Sigma) overnight at 4°C. The tissues were then decalcified in 0.5 M EDTA (pH 8.0, ethylenediaminetetraacetic acid) for up to seven days and infiltrated in a series of sucrose solutions before being embedded in OCT. The frozen tissues were cut into 20 µm coronal sections on a cryostat. Mice used in immunohistochemistry were intraperitoneally injected with two doses of 5-bromo-2′-deoxyuridine (BrdU), each at 50 mg/kg body weight followed by a two-hour waiting period before being sacrificed. The procedures of animal handling and tissue harvesting were approved by the institutional animal care and use committee of the University of Pennsylvania.

### *In situ* Hybridization and Cell Counting

The procedure for *in situ* hybridization was published previously [31], [32]. Briefly, digoxigenin (DIG) labeled RNA probes of the OR genes were generated using DIG RNA Labeling Kit (SP6/T7) (Roche, Indianapolis, IN). The primers for cloning the 15 OR genes are included in the Table S1. All OR probes presumably detected mRNAs from a single OR gene with one exception, MOR270-1. This probe stained mRNAs from both MOR270-1 and MOR271-1 which share 98% of nucleotide homology [31].

The sections were hybridized with the RNA probes (∼1 µg/ml) overnight at 65°C in the hybridization solution (50% deionized formamide, 10 mM Tris-Cl (pH 8.0), 10% dextran sulfate, 1X Denhardt’s solution, 200 µg/ml tRNA, 0.6 M NaCl, 0.25% SDS and 1 mM EDTA), followed by high-stringency washing steps sequentially in 2x, 0.2x and 0.1x SSC at 65°C (20 min in each solution). The sections were then incubated with alkaline phosphatase (AP)-conjugated anti-DIG antibody (Anti-digoxigenin-AP, Roche) at room temperature (RT) for 1 hr. The signals were detected after 2 hr incubation at RT in solution of nitro blue tetrazolium and 5-bromo-4-chloro-3-indolyl phosphate (NBT/BCIP, Roche). NBT/BCIP stained cells appeared as dark brown dots in the tissue sections, and most dots (85–90%) in the mature OSN layer were unequivocally individual cells with a characteristic contour. A questionable dot was counted only if it met the following two criteria: its diameter was greater than 4 µm (half of the average diameter of mature OSNs) and its intensity exceeded 30% of the darkest cell on that section [32]. The potential overcounting problem was not corrected because the cell density for all OR genes was calculated and compared in the same way.

**Figure 1:**
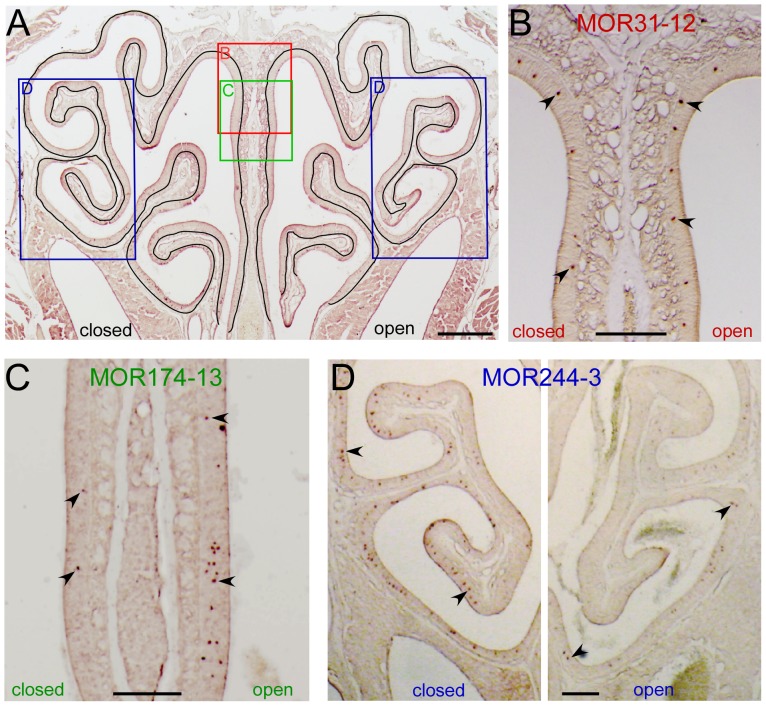
Unilateral naris closure differentially alters the cell density of individual OR genes. (A) The linear length from a coronal section was determined by outlining the basement membrane which separates the olfactory epithelium from the propria lamina. The rectangles indicate the approximate locations from which images in B–D were taken. (B–D) The coronal sections were hybridized with antisense RNA probes of MOR13-4 (dorsal zone, B), MOR174-13 (intermediate zone, C), and MOR244-3 (ventral zone, D). The two images in D were taken under identical conditions from the same section and the staining in the open side appeared much weaker than that in the closed side. Arrows mark examples of labeled cells. Scale bar = 0.5 mm in A and 0.2 mm in B–D.

To minimize variations from the tissue source, we used approximately 30 coronal sections (∼ 600 µm) from each animal, collected from the middle to the posterior part of the nose. These sections had characteristic morphology as shown in Fig. 1A. Adjacent sections from each animal were tested for different OR probes. A single OR probe was typically tested on one or two sections (200–300 µm apart) from a single animal and each section was therefore treated as an independent sample. For each section, the linear length of the main olfactory epithelium was measured by outlining the basement membrane that separates the olfactory epithelium from the propria lamina using Canvas 12 (Deneba) (Fig. 1A). Because there was no definite boundary between different zones, we used the linear length of the entire closed or open side to calculate the cell density for each OR gene in the naris-closed animals. For wild-type and OMP-Kir2.1 mice, the cell density from each section was averaged from the two sides.

### Immunohistochemistry

**Figure 2:**
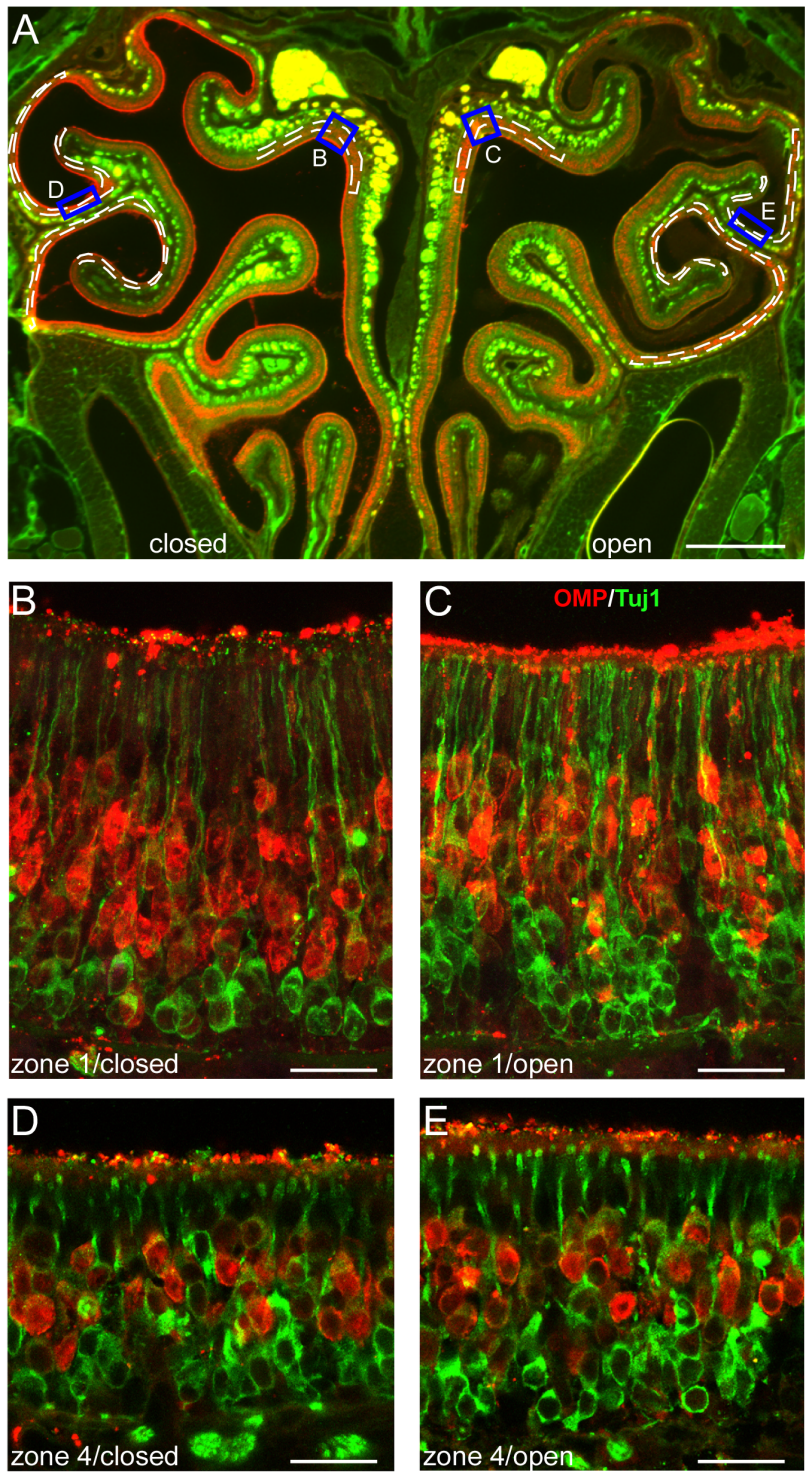
The thickness of mature OSN layer does not differ significantly between the closed and open side. Coronal sections from 4-week-old mice that underwent neonatal, unilateral naris closure were stained with antibodies against OMP (red) and a neuronal marker Tuj1 (green). (A) A low-magnification image shows an entire section. Scale bar = 0.5 mm. The rectangles indicate the approximate locations where confocal images in B to E were taken. The dashed lines illustrate how the thickness of the mature OSN layer is measured in defined regions of zone 1 and zone 4. (B–E) High-magnification confocal images (projected from a stack of 5 images with z step = 3 µm) were taken from zone 1 and zone 4 in the closed (B, D) and open side (C, E). Scale bars = 20 µm.

After antigen retrieval in a 95°C waterbath for 10 min (followed by 10 min in 2N HCl at 37°C for BrdU detection), the tissue sections were blocked for 60 min in 0.3% Triton X-100 in phosphate-buffered saline with 3% bovine serum albumin, and then incubated at 4°C with the primary antibodies in the same solution overnight. The primary antibodies included chicken anti-OMP (1∶500, a generous gift from Dr. Qizhi Gong, University of California, Davis), mouse anti-β3 Tubulin Antibody (Tuj1, 1∶200, sc-58888, Santa Cruz), rabbit anti-cleaved caspase-3 (1∶200, Catalog #9661, Cell Signaling), and mouse anti-BrdU (1∶1000, MAB3222, Chemicon International). Immunofluorescence was achieved by reaction with appropriate secondary antibodies at 1∶200 for 1 h. The secondary antibodies (from Molecular Probes, Invitrogen) included donkey anti-mouse-488 (A21202), donkey anti-rabbit-568 (A10042), and goat anti-chicken-647 (A21449). Tissues were washed in 0.3% Triton X-100 in phosphate-buffered saline, and mounted in Vectashield (Vector Laboratories). Fluorescent images were taken under a SP5/Leica confocal microscope with LAS AF Lite software. To quantify the immunohistochemistry data, we standardized the measurement and counting across all sections and the data for zone 1 and zone 4 were obtained from the dashed mediodorsal and lateroventral regions (Fig. 2A), respectively. The thickness of mature OSN layer was determined as the area of OMP-positive layer divided by the linear length. BrdU and caspase-3 positive cells were readily counted in high-magnification confocal images and the cell density was calculated as the cell number divided by the liner length. For wild-type and OMP-Kir2.1 mice, the data for each section were averaged from the two sides.

### Data Analysis

All averaged data are presented as mean ± standard error (*n* = number of sections). For naris-closed mice, the data from the closed and open side were compared by paired t-tests. For OMP-Kir2.1 mice and wild-type controls (grouped from littermate Kir2.1 negative and wild-type mice), unpaired two-tailed tests were used for in situ hybridization data. Multiple comparison correction is not applied because each OR was tested in a distinct set of sections and treated as an independent study. Two-way ANOVA tests were used to compare the thickness of mature OSN layer and the density of BrdU and caspase-3 positive cells from different zones (zone 1 or zone 4) and from different animals (wild-type or OMP-Kir2.1). Statistical tests were performed using Excel and Prism Graphpad.

## Results

### Unilateral Naris Closure Differentially Alters the Cell Density of Individual OR Genes

To investigate how sensory experience modulates OR gene expression in the olfactory epithelium, we performed unilateral naris closure on neonatal mice and examined the mRNA expression patterns for individual OR genes four weeks later via *in situ* hybridization. Fifteen OR genes from different zones were selected because their antisense probes generated strong staining signals in the olfactory epithelium (Fig. 1). Recent studies have convincingly demonstrated that ORs are expressed in shifting and overlapping stripes rather than originally-defined four zones [5], [8]. However, for the simplicity of description, we grouped these 15 genes into three “zones” by referring to the original definition (mediodorsal zone 1, intermediate zone 2–3, and lateroventral zone 4). We compared the cell density (represented as the cell number per linear length in mm, see Materials and Methods for details) for each OR gene between the closed and open side (Fig. 1A). Among the 15 OR genes, we found all three possible patterns after unilateral naris closure (Table 1). Pattern 1 (closed = open; the cell density is similar between the two sides) was observed for three ORs (Fig. 1B). Pattern 2 (closed<open; the cell density in the closed side is significantly lower than that in the open side) was observed for four ORs (Fig. 1C). Pattern 3 (closed>open; the cell density in the closed side is significantly higher than that in the open side) was observed for the remaining eight ORs (Fig. 1D). These results indicate that unilateral naris closure has differential effects on the expression of individual OR genes.

**Table 1:** Naris closure causes differential changes in the cell density for different OR genes.

Zone	OR Gene	Cell Density (cell number/mm)		
		Closed	Open	Paired *t* test
Mediodorsal zone 1	**Olfr640 (MOR13-4)**	**18.49±2.25**	**14.17±2.37 (n = 9)**	**p<0.05**
	Olfr648 (MOR31-12)	0.57±0.09	0.66±0.06 (n = 6)	p>0.05
	*Olfr663 (MOR40-12)*	*0.34±0.08*	*0.41±0.08 (n = 7)*	*p<0.05*
Intermediate zone 2-3	Olfr1168 (MOR174-13)*	*1.04±0.13*	*1.81±0.08 (n = 6)*	*p<0.01*
	Olfr10 (MOR256-55 or L45)	6.88±0.71	7.25±0.88 (n = 9)	p>0.05
	Olfr164 (MOR279-2)	0.65±0.17	0.71±0.17 (n = 8)	p>0.05
	*Olfr17 (MOR263-5 or P2)*	*2.49±0.23*	*3.27±0.25 (n = 9)*	*p<0.001*
	*Olfr142 (MOR227-2 or K20)*	*3.11±0.36*	*4.44±0.37 (n = 8)*	*p<0.01*
Lateroventral zone 4	**Olfr166 (MOR270-1)**	**13.65±1.05**	**11.59±1.22 (n = 8)**	**p<0.05**
	Olfr455 (MOR0-2)**	**7.01±0.73**	**4.64±0.82 (n = 8)**	**p<0.001**
	**Olfr1509 (MOR244-3)**	**6.69±0.45**	**4.45±0.36 (n = 8)**	**p<0.01**
	**Olfr1264 (MOR236-1)**	**5.73±0.47**	**4.41±0.40 (n = 8)**	**p<0.001**
	**Olfr1260 (MOR232-2)**	**4.61±0.60**	**3.11±0.42 (n = 8)**	**p<0.05**
	**Olfr140 (MOR235-1)**	**4.56±0.53**	**3.57±0.41 (n = 8)**	**p<0.01**
	**Olfr281 (MOR160-5)**	**4.00±0.23**	**2.38±0.30 (n = 8)**	**p<0.01**

The averaged cell density per linear length is denoted as cell number ± s.e.m./mm (*n* = number of sections). Pattern 1 (similar in both sides) is in regular script, Pattern 2 (closed<open) in *italic*, and Pattern 3 (closed>open) in **bold**. * Cells expressing MOR174-13 were found in both the mediodorsal and intermediate zones. ** Cells expressing MOR0-2 were found in both the intermediate and lateroventral zones.

Curiously, all seven OR genes in the lateroventral zone showed Pattern 3 (closed>open), whereas the eight OR genes in the mediodorsal or intermediate zones displayed all three patterns. There are potentially three possible scenarios underlying this observation. First, Pattern 3 (closed>open) applies to most if not all OR genes expressed in the lateroventral zone 4, which would lead to the prediction that the total number of OR-expressing OSNs in this zone is higher in the closed side than in the open side. To test this possibility, we compared the thickness of mature OSN layer in the closed and open side via immunohistochemistry. Confirming the previous reports [13], [14], [15], [21], we observed more layers of immature OSNs in the open side presumably due to increased neurogenesis (Fig. 2A–E). In the lateroventral zone 4 (dashed areas in Fig. 2A), the thickness of mature OSN layer was similar between the closed (26.30±0.56 µm) and open side (25.05±0.76 µm, n = 17 sections, p = 0.134 in paired, two-tailed *t* test). Because the size of individual cells, the density of OSNs, and the linear length of the olfactory epithelium are indistinguishable between the two sides (Fig. 2), these results suggest that the total number of mature OSNs is similar in the lateroventral zone between the two sides and rule out the first possibility. It should be noted that this does not rule out that the number of OSNs expressing some OR types is higher in the closed side than that in the open side.

Second, the mRNA level in individual OSNs is lower in the open side, which makes some of the stained cells fall below the detection threshold of in situ hybridization. Indeed, the signals of stained cells in the open side often appeared weaker than those in the closed side (Fig. 1D). Third, the tested seven OR genes represent only a small fraction expressed in the lateroventral zone. It is possible that other untested ORs in the ventral zone show Pattern 2 (closed<open), therefore the changes in cell densities of different ORs keep the total number of OSNs balanced between the closed and open side. To distinguish the second and third possibility, we analyzed the data from a recent study, in which the expression level of individual OR genes (a total of 701) was compared between the closed and open side using the Mouse Whole Genome Microarray [19]. We summarized the microarray data for individual OR genes whose zonal distributions have been determined by *in situ* hybridization [6], [8], [9], [31], [33], [34], [35], [36], [37], [38], [39], [40]. Out of 14 OR genes expressed in the lateroventral zone, 12 showed a log2(x/o) ratio (closed/open) between 0.28 and 0.88 suggesting the expression level in the closed side is 21.4% to 84.0% higher than that in the open side and the difference for seven ORs reached statistical significance (p<0.05, Table 2). Another OR (Olfr281) had a log2 ratio of 0.15 and the remaining one (Olfr535) showed a close-to-zero log2 ratio suggesting the same expression level between the closed and open side. Not a single OR gene displayed a negative log2 ratio which would imply Pattern 2 (closed<open). These data lend support to the second possibility in which the mRNA level in individual OSNs is higher in the closed side than in the open side; even though it does not completely rule out the third possibility, which may contribute to a lesser extent (see Discussion). Among the 24 OR genes expressed in the mediodorsal or intermediate zones, the expression level was significantly higher (for ten ORs), similar (for 13 ORs), or significantly lower (for one OR) in the closed side as compared to that in the open side. Taken together, these results indicate that sensory experience during the first postnatal month exerts differential effects on the expression of individual OR genes.

**Table 2:** Naris closure induces higher expression levels in the closed side for most OR genes expressed in the lateroventral zone 4.

OR Gene	Log2 ratio (x/o)	P value
**Olf370 (MOR267-16)**	**0.88043**	**1.76E-06**
**Olfr1282 (MOR248-2)**	**0.8062514**	**1.65E-05**
**Olfr140 (MOR235-1)**	**0.6121188**	**0.000161526**
**Olfr398 (MOR157-1)**	**0.5384432**	**0.018259043**
**Olfr6 (MOR103-16 or M50)**	**0.494558**	**0.001896377**
**Olfr15 (MOR256-17)**	**0.483775**	**0.015592738**
Olfr2 (MOR103-15 or I7)	0.3563172	0.168610275
**Olfr166 (MOR270-1)**	**0.3376114**	**0.00183897**
Olfr1260 (MOR232-2)	0.3243242	0.130942687
Olfr1509 (MOR244-3 or MOR83)	0.2998145	0.312687695
Olfr1508 (MOR244-2 or Mor10)	0.2907011	0.260114938
Olfr1507 (MOR244-1 or Mor28)	0.2847244	0.258988827
Olfr281 (MOR160-5)	0.1466437	0.4801853
Olfr535 (MOR253-7)	−0.0369031	0.901128709

Log2 ratio (x/o), the expression level ratio between the closed (x) and open (o) side, and the p value were obtained from the microarray study [19]. Pattern 1 (similar in both sides) is in regular script and Pattern 3 (closed>open) in **bold**. The OR genes are sorted based on the log2 ratio and only significant changes (p<0.05) are in **bold**.

### Decreasing OSN Excitability Reduces the Cell Density for Most OR Genes

OSN activation involves several sequential steps: odorant binding activates specific OR proteins, which trigger the signal transduction cascade leading to depolarization and subsequently spike firing in these neurons. To understand the role of sensory inputs and neuronal activity in shaping OR expression, we would need mouse models that can separate these two processes. The unilateral naris-occluded mice studied above have altered sensory experience in the closed and open nostril, but OSN excitability presumably remains normal. We next performed similar OR expression analysis in transgenic OMP-Kir2.1 mice in which mature OSNs overexpress the inward rectifying potassium channel Kir2.1 [25]. In contrast to unilateral naris closure, OMP-Kir2.1 mice have normal sensory experience but significantly reduced neuronal activity in OSNs.

**Figure 3:**
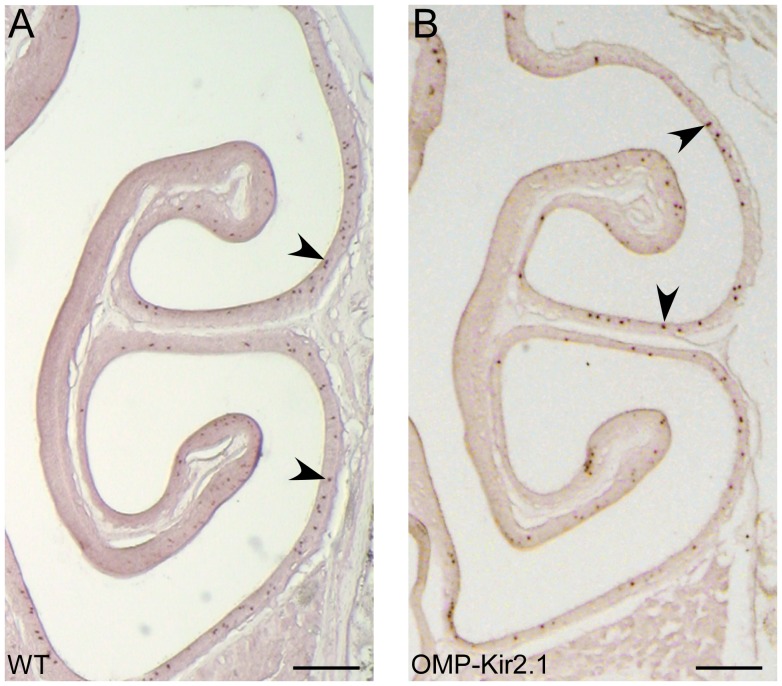
The MOR236-1 cell density is lower in OMP-Kir2.1 mice as compared with wild-type mice. The coronal sections from wild-type (A) and OMP-Kir2.1 (B) mice were hybridized with an antisense probe for MOR236-1 gene. Arrows mark examples of labeled cells. Scale bars = 0.2 mm.

**Figure 4:**
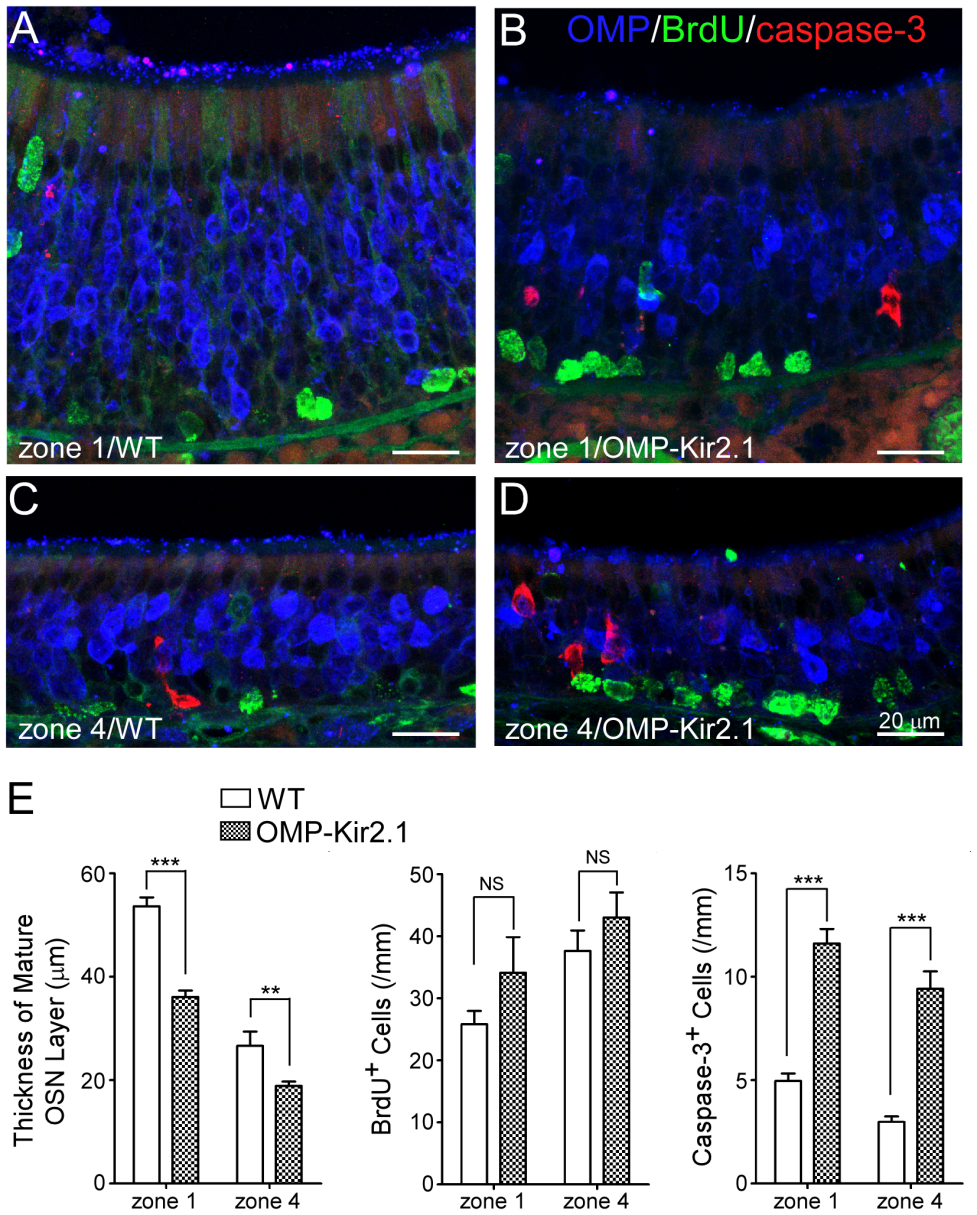
OMP-Kir2.1 mice have thinner olfactory epithelium with an increased density of apoptotic cells than wild-type controls. (A–D) Coronal sections from wild-type and OMP-Kir2.1 mice were stained with antibodies against OMP (blue), BrdU (green) and cleaved caspase-3 (red). Confocal images (projected from a stack of 5 images with z step = 3 µm) were taken from zone 1 and zone 4 in wild-type (A, C) and OMP-Kir2.1 (B, D) mice. (E) Summary of the thickness of mature OSN layer and the density of BrdU and caspase-3 positive cells in zone 1 and zone 4 from wild-type and OMP-Kir2.1 mice. Statistical significance was obtained by Bonferroni post-hoc tests: NS (not significant) indicates p>0.05, ** p<0.01, and *** p<0.001.

We selected seven ORs which displayed different patterns after naris closure (Table 1): Olfr10 and Olfr164 (Pattern 1), Olfr17 and Olfr142 (Pattern 2), and Olfr1264, Olfr1260, and Olfr281 (Pattern 3). We compared the cell density for these seven ORs in OMP-Kir2.1 mice versus wild-type controls and one example is shown in Fig. 3. Regardless of the zonal distribution or the expression pattern change in the naris-closed mice, the cell density for all seven ORs tested is reduced in OMP-Kir2.1 mice as compared with the wild-type controls. The reduction ranges from 12.8% to 54.2% and the difference for all ORs except Olfr1260 reaches statistical significance (Table 3). If the cell density reduction applies to most OR genes, we would predict that the total number of OSNs in OMP-Kir2.1 mice is lower than that of control mice. To test this possibility, we measured the thickness of mature OSN layer in defined regions of the olfactory epithelial sections stained with the OMP antibody (Fig. 4A–E). The mature OSN layer was significantly thinner in OMP-Kir2.1 mice (n = 17 sections) than that in the wild-type mice (n = 10) in both the mediodorsal zone 1 (36.10±1.22 µm in OMP-Kir2.1 vs 53.64±1.68 µm in WT, p<0.001) and the lateroventral zone 4 (18.88±0.84 µm in OMP-Kir2.1 vs 26.62±2.74 µm in WT, p<0.01, Bonferroni post-hoc tests) (Fig. 4E), supporting that there is a reduction of mature OSNs in OMP-Kir2.1 mice.

**Table 3:** The cell density for most OR types decreases in OMP-Kir2.1 mice.

OR Gene	Cell Density (cell number/mm)				
	OMP-Kir2.1	Wild-type	*t* test	Reduction	
Olfr10 (MOR256-55 or L45)	2.80±0.41 (n = 6)	6.11±1.95 (n = 11)	p<0.001		54.2%
Olfr164 (MOR279-2)	0.50±0.03 (n = 12)	1.08±0.11 (n = 12)	p<0.001		53.7%
Olfr1264 (MOR236-1)	3.06±0.41 (n = 7)	4.78±0.33 (n = 10)	p<0.01		36.0%
Olfr142 (MOR227-2 or K20)	2.50±0.33 (n = 7)	3.68±0.20 (n = 10)	p<0.05		32.1%
Olfr281 (MOR160-5)	2.27±0.29 (n = 6)	3.19±0.23 (n = 10)	p<0.05		28.8%
Olfr17 (MOR263-5 or P2)	1.51±0.05 (n = 8)	2.08±0.19 (n = 12)	p<0.05		27.4%
Olfr1260 (MOR232-2)	2.85±0.49 (n = 9)	3.27±0.30 (n = 10)	p = 0.48		12.8%

The averaged cell density per linear length is denoted as cell number ± s.e.m./mm (*n* = number of sections). The wild-type data were combined from littermate controls (Kir2.1 negative) and untreated C57Bl/6 mice. The p values were obtained by unpaired, two-tailed *t* tests.

The thinner olfactory epithelium observed in OMP-Kir2.1 mice could be caused by decreased neurogenesis and/or increased apoptosis. To differentiate these two potential mechanisms, we stained the olfactory epithelium with BrdU and cleaved caspase 3 to label proliferating and apoptotic cells, respectively. In wild-type animals, the density of BrdU positive cells was significantly higher in the lateroventral zone 4 (37.63±3.31/mm linear length, n = 17 sections) than in the mediodorsal zone 1 (25.81±2.16/mm, n = 19), consistent with a previous report [41]. The zonal difference was also observed in OMP-Kir2.1 mice (43.04±4.01/mm in zone 4, n = 17 and 34.09±5.77/mm in zone 1, n = 11). The difference between different zones was significant (F = 7.84, p<0.01), but the difference between animals (OMP-Kir2.1 vs WT) was not (F = 3.41, p = 0.0699; two-way ANOVA) (Fig. 4E). Similarly we compared the density of caspase-3 positive cells between wild-type (2.98±0.26/mm in zone 4, n = 17 and 4.96±0.37/mm in zone 1, n = 19) and OMP-Kir2.1 mice (9.41±0.84/mm in zone 4, n = 21 and 11.61±0.70/mm in zone 1, n = 17). Two-way ANOVA revealed that the density of caspase-3 positive cells was significantly higher in OMP-Kir2.1 (F = 111.9, p<0.0001) than wild-type mice and the density was also significantly higher in zone 1 than in zone 4 (F = 11.35, p = 0.0012) (Fig. 4E). Therefore, reduction of OSN excitability leads to a decrease in the number of mature OSNs by increasing apoptotic cells, suggesting that neuronal activity is critical for survival of OSNs.

## Discussion

By manipulating the sensory inputs and neuronal excitability, we investigated activity-dependent modulation of OR gene expression in the mouse olfactory epithelium using in situ hybridization. We found that altering the sensory inputs via unilateral naris closure has differential effects on individual OR genes: the expression level in the closed side can be significantly lower, similar, or significantly higher as compared to that in the open side. In contrast, decreasing the excitability of OSNs in OMP-Kir2.1 mice leads to reduced cell densities for most OR genes and thinner olfactory epithelium with an increased density of apoptotic cells, supporting that neuronal activity is critical for survival of OSNs. Our results suggest that early experience can shape OR gene expression patterns, which are modulated distinctively by sensory inputs and neuronal activity.

Unilateral naris closure causes differential changes in the expression patterns of individual OR genes (Fig. 1 and Table 1). In fact, all three possible patterns have been observed; i.e., the cell density in the closed can be lower, similar or higher as compared to that in the open side. This finding may result from one or more of the following potential mechanisms. First, different ORs have distinct functions and are differentially stimulated in early postnatal life. One can imagine that OSNs expressing ORs essential for suckling are fully functional and properly stimulated by olfactory cues right after birth, while those expressing ORs essential for mating may not be functional and/or stimulated before the animals become sexually mature. Because the environmental odors do not equally stimulate all ORs, deprivation of sensory inputs in the closed side or overstimulation in the open side will cause differential effects on individual OR genes. Odor stimulation has been shown to increase the lifespan of OSNs by activating MAPK/CREB-dependent transcriptional pathway [42] and/or by inhibiting the expression of a replication-independent histone variant H2BE, which promotes neuronal cell death [43]. Interestingly, OR- and activity-dependent expression of H2BE in OSNs may contribute to differential modulation of OR expression patterns after naris-closure [43]. Second, OSNs expressing different ORs show asynchronous temporal onset and reach their maximum density at different stages ranging from embryonic days to postnatal one month [32], [44]. Neonatal naris closure for four weeks may have more significant effects on OSNs expressing a “late” OR gene than those expressing an “early” one. Third, unilateral naris closure not only alters the odor inputs to the two nostrils but also changes the mechanical stimulation carried by the air flow. A subset of OSNs in the mouse olfactory epithelium can respond to mechanical stimulation in addition to odors [45]. OSNs expressing certain receptors are more sensitive to mechanical stimulation than those expressing others (our unpublished data), suggesting that they may be differently affected by naris closure. Fourth, OSNs located in different zones may be subject to different modulation by sensory inputs. In fact, the mediodorsal zone and lateroventral zone have different cell proliferation rate and epithelial thickness (Fig. 4) [41]. We found that all seven OR genes expressed in the lateroventral zone show the same pattern (closed>open) while the other eight ORs in the mediodorsal and intermediate zone are heterogeneous (Table 1), suggesting the olfactory epithelium in different zones are differentially regulated by activity. Further investigation would be required to determine to what extent each of these mechanisms contributes to the differential modulation of OR gene expression.

A recent study using microarrays to examine the expression level of individual OR genes reveals differential regulation: the expression level can be higher, similar, or lower in the closed side than in the open side [19]. Among the fifteen ORs we tested in Table 1, ten are detected in the microarray study. All ten ORs showed the same trend in these two studies, with four of them (Olfr640, Olfr648, Olfr166, and Olfr140) reaching comparable statistical significance. For the other six OR genes (Olfr1168, Olfr164, Olfr17, Olfr1509, Olfr1260, and Olfr281), the difference between the closed and open side did not reach statistical significance in either our study (for Olfr164) or in the microarray study (for the other five ORs). The discrepancy between these two studies is likely caused by methodological factors. These two methods may have different sensitivities for individual OR genes and the environments for the experimental animals may be different. In addition, microarrays examine the expression level of individual ORs from the entire olfactory mucosa while in situ hybridization reveals individual OSNs expressing an OR gene in selected tissue sections. For example, the following two scenarios would generate the same outcome in microarray analysis but different outcomes by in situ hybridization: (1) the total number of OSNs expressing a particular receptor is reduced by 50% but the expression level in each cell stays the same; and (2) the total number of OSNs stays the same but the expression level in individual cells is reduced by 50%. To fully understand the expression level/pattern changes of a single gene, it is essential to employ multiple, complementary approaches. By examining genetically labeled OSNs in P2-IRES-tauLaz mice, two groups reported that sensory deprivation significantly reduced the number of P2 cells [24], [46], which is confirmed by our in situ hybridization data. It is worth noting that both in situ hybridization and microarrays examine the mRNA expression, and more studies would be required to understand how OR protein expression is modulated.

In contrast to unilateral naris closure which alters the sensory inputs into the two nostrils, OMP-Kir2.1 mice have decreased spontaneous and sensory-evoked activities in mature OSNs without changing the sensory inputs [25]. Decreased excitability of OSNs leads to a reduced cell density for six out of seven ORs regardless of their change patterns in unilateral naris-occluded mice (Fig. 3 and Table 3). We further demonstrated that the olfactory epithelium in OMP-Kir2.1 mice is significantly thinner with an increased density of apoptotic cells as compared with the wild-type controls (Fig. 4), indicating that neuronal activity is essential for survival of OSNs. It has been shown that the spontaneous neuronal activity is required for the establishment and maintenance of the peripheral sensory map from OSNs to the olfactory bulb [25]. It is possible that neuronal activity of OSNs triggers the release of some neurotrophic factors from the olfactory bulb promoting prolonged survival of OSNs [47], [48]. Because OSNs expressing different OR types show different spontaneous firing rates [43] and may not be equally stimulated by the environmental odors, it is therefore conceivable that decreasing neuronal excitability leads to a reduction in cell densities for all OR types but to different extent (Table 3). This may explain why sensory deprivation in the closed side does not cause the same effect as in OMP-Kir2.1 mice because the OSNs in the deprived side nevertheless keep their spontaneous firing capability. Our results indicate that OR gene expression is subject to activity-dependent modulation, but sensory inputs and neuronal activity may play distinct roles in shaping the OR expression patterns.

## Supporting Information

Table S1 **The following primers were used to clone the 15 OR genes for generating the antisense RNA probes.**
(DOCX)
