# Supplementary material for: Activity-Dependent Modulation of Odorant Receptor Gene Expression in the Mouse Olfactory Epithelium
Source: PLoS One. 2013 Jul 29;8(7):e69862. doi: 10.1371/journal.pone.0069862 (PMC3726745; doi:10.1371/journal.pone.0069862)
Supplement: Table S1 — The following primers were used to clone the 15 OR genes for generating the antisense RNA probes. (DOCX) [file pone.0069862.s001.docx]

**Table S1.**

**1. MOR13-4 (Olfr640)** (accession number: AY073271, coding region = 945 bp; probe length = 522 bp)

Degenerate primers

Forward primer: ATG GCI T(T/A)(T/C) GA(T/C) (C/A)GI T(T/A)(T/C) (T/C/G)TI GC

Reverse primer: AT IA(A/T/G) IGG (G/A)TT IA(A/G) CAT

**2. MOR31-12 (Olfr648)** (accession number: NM_146751, coding region = 951 bp; probe length = 410 bp)

Forward primer: AATGTGAGCAGTTACAACCCG

Reverse primer: TTGGGGGTCAAGATAGTAGCA

**3. MOR40-12 (Olfr663)** (accession number: NM_001011757, coding region = 1035 bp; probe length = 528 bp)

Degenerate primers

Forward primer: ATG GCI T(T/A)(T/C) GA(T/C) (C/A)GI T(T/A)(T/C) (T/C/G)TI GC

Reverse primer: AT IA(A/T/G) IGG (G/A)TT IA(A/G) CAT

**4. MOR174-13** **(Olfr1168)** (accession number: NM_146531, coding region = 936 bp; probe length = 513 bp)

Degenerate primers

Forward primer: ATG GCI T(T/A)(T/C) GA(T/C) (C/A)GI T(T/A)(T/C) (T/C/G)TI GC

Reverse primer: AT IA(A/T/G) IGG (G/A)TT IA(A/G) CAT

**5. MOR256-55 (Olfr10, L45)** (accession number: NM_206822, coding region = 936 bp; probe length = 919 bp)

Forward primer: TATCAGTTTGGGAGGAGGCTT

Reverse primer: CCCTGTGACTGTAGCTCTTCC

**6. MOR279-2 (Olfr164)** (accession number: NM_146451, coding region = 948 bp; probe length = 494 bp)

Forward primer: GCTATTTGCTACCCATTAAGG

Reverse primer: ATGAGGGGGTTCAACATTG

**7. MOR263-5 (Olfr17, P2)** (accession number: NM_020598, coding region = 948 bp; probe length = 935 bp)

Forward primer: ACTGGACAACTGTCAGGGAAT

Reverse primer: TCATAGTTTCTGAGGGCCCA

**8. MOR227-2 (Olfr142, K20)** (accession number: NM_146984, coding region = 918 bp; probe length = 818 bp)

Forward primer: TTTCCAGGATCCAGAGGTTCA

Reverse primer: TATAGACAATGGGGTTCAGCA

**9. MOR270-1 (Olfr166)** (accession number: NM_147068, coding region = 939 bp; probe length = 513 bp)

Degenerate primers

Forward primer: ATG GCI T(T/A)(T/C) GA(T/C) (C/A)GI T(T/A)(T/C) (T/C/G)TI GC

Reverse primer: AT IA(A/T/G) IGG (G/A)TT IA(A/G) CAT

**10. MOR0-2 (Olfr455)** (accession number: NM_001081301, coding region = 954 bp; probe length = 403 bp)

Forward primer: TTCCTTTGCCAGTTGTCAGC

Reverse primer: TCCGTGTACAGGCCAAGTG

**11. MOR244-3 (Olfr1509)** (accession number: NM_020514, coding region = 1010 bp; probe length = 520 bp)

Forward-Primer: GGGAACTTCCTCATTGTTGTT

Reverse-Primer: AGGCTAGAAAACATACGAGGG

**12. MOR236-1 (Olfr1264)** (accession number: NM_021368, coding region = 927 bp; probe length = 504 bp)

Degenerate primers

Forward primer: ATG GCI T(T/A)(T/C) GA(T/C) (C/A)GI T(T/A)(T/C) (T/C/G)TI GC

Reverse primer: AT IA(A/T/G) IGG (G/A)TT IA(A/G) CAT

**13. MOR232-2 (Olfr1260)** (accession number: NM_146981, coding region = 933 bp; probe length = 504 bp)

Degenerate primers

Forward primer: ATG GCI T(T/A)(T/C) GA(T/C) (C/A)GI T(T/A)(T/C) (T/C/G)TI GC

Reverse primer: AT IA(A/T/G) IGG (G/A)TT IA(A/G) CAT

**14. MOR235-1 (Olfr140)** (accession number: NM_020515, coding region = 909 bp; probe length = 504 bp)

Degenerate primers

Forward primer: ATG GCI T(T/A)(T/C) GA(T/C) (C/A)GI T(T/A)(T/C) (T/C/G)TI GC

Reverse primer: AT IA(A/T/G) IGG (G/A)TT IA(A/G) CAT

**15. MOR160-5 (Olfr281)** (accession number: NM_146280, coding region = 936 bp; probe length = 519 bp)

Forward-Primer: AACCTGACGATGCTGCTGGT

Reverse-Primer: GATTGGGAGAAAGGTCCCC
